# Supplementary material for: Pyruvate Dehydrogenase Kinase 1 inhibition mediated oxidative phosphorylation enhancement in cartilage promotes osteoarthritis progression
Source: BMC Musculoskelet Disord. 2023 Jul 20;24:597. doi: 10.1186/s12891-023-06585-6 (PMC10357736; doi:10.1186/s12891-023-06585-6)
Supplement: Supplementary file 1 — Additional file 1. [file 12891_2023_6585_MOESM1_ESM.zip › MF.Gsea.1653627316242/GOMF_PHOSPHOTRANSFERASE_ACTIVITY_PHOSPHATE_GROUP_AS_ACCEPTOR.html]

Details for gene set GOMF\_PHOSPHOTRANSFERASE\_ACTIVITY\_PHOSPHATE\_GROUP\_AS\_ACCEPTOR[GSEA]

|  || Dataset | 55457\_collapsed\_to\_symbols.cls.cls #OA\_versus\_normal.cls.cls #OA\_versus\_normal\_repos |
| Phenotype | cls.cls#OA\_versus\_normal\_repos |
| Upregulated in class | OA |
| GeneSet | GOMF\_PHOSPHOTRANSFERASE\_ACTIVITY\_PHOSPHATE\_GROUP\_AS\_ACCEPTOR |
| Enrichment Score (ES) | 0.46265653 |
| Normalized Enrichment Score (NES) | 1.6199154 |
| Nominal p-value | 0.012437811 |
| FDR q-value | 0.13372667 |
| FWER p-Value | 0.974 |
Table: GSEA Results Summary

  

Fig 1: Enrichment plot: GOMF\_PHOSPHOTRANSFERASE\_ACTIVITY\_PHOSPHATE\_GROUP\_AS\_ACCEPTOR      
 Profile of the Running ES Score & Positions of GeneSet Members on the Rank Ordered List

  

| SYMBOL | TITLE | RANK IN GENE LIST | RANK METRIC SCORE | RUNNING ES | CORE ENRICHMENT || 1 | NME7 | NME/NM23 family member 7 [Source:HGNC Symbol;Acc:HGNC:20461] | 178 | 0.835 | 0.0800 | Yes |
| 2 | PMVK | phosphomevalonate kinase [Source:HGNC Symbol;Acc:HGNC:9141] | 346 | 0.725 | 0.1485 | Yes |
| 3 | NME5 | NME/NM23 family member 5 [Source:HGNC Symbol;Acc:HGNC:7853] | 362 | 0.720 | 0.2282 | Yes |
| 4 | AK1 | adenylate kinase 1 [Source:HGNC Symbol;Acc:HGNC:361] | 481 | 0.667 | 0.2939 | Yes |
| 5 | NME4 | NME/NM23 nucleoside diphosphate kinase 4 [Source:HGNC Symbol;Acc:HGNC:7852] | 677 | 0.599 | 0.3460 | Yes |
| 6 | NME3 | NME/NM23 nucleoside diphosphate kinase 3 [Source:HGNC Symbol;Acc:HGNC:7851] | 876 | 0.549 | 0.3924 | Yes |
| 7 | NME2 | NME/NM23 nucleoside diphosphate kinase 2 [Source:HGNC Symbol;Acc:HGNC:7850] | 1428 | 0.439 | 0.3989 | Yes |
| 8 | GUK1 | guanylate kinase 1 [Source:HGNC Symbol;Acc:HGNC:4693] | 1640 | 0.407 | 0.4283 | Yes |
| 9 | MPP1 | membrane palmitoylated protein 1 [Source:HGNC Symbol;Acc:HGNC:7219] | 1761 | 0.389 | 0.4627 | Yes |
| 10 | CASK | calcium/calmodulin dependent serine protein kinase [Source:HGNC Symbol;Acc:HGNC:1497] | 2316 | 0.324 | 0.4561 | No |
| 11 | DLG2 | discs large MAGUK scaffold protein 2 [Source:HGNC Symbol;Acc:HGNC:2901] | 2768 | 0.273 | 0.4517 | No |
| 12 | ITPKB | inositol-trisphosphate 3-kinase B [Source:HGNC Symbol;Acc:HGNC:6179] | 3467 | 0.205 | 0.4206 | No |
| 13 | DTYMK | deoxythymidylate kinase [Source:HGNC Symbol;Acc:HGNC:3061] | 3538 | 0.199 | 0.4375 | No |
| 14 | AK5 | adenylate kinase 5 [Source:HGNC Symbol;Acc:HGNC:365] | 3841 | 0.172 | 0.4333 | No |
| 15 | PPIP5K2 | diphosphoinositol pentakisphosphate kinase 2 [Source:HGNC Symbol;Acc:HGNC:29035] | 3986 | 0.157 | 0.4398 | No |
| 16 | AK2 | adenylate kinase 2 [Source:HGNC Symbol;Acc:HGNC:362] | 4179 | 0.140 | 0.4406 | No |
| 17 | NME6 | NME/NM23 nucleoside diphosphate kinase 6 [Source:HGNC Symbol;Acc:HGNC:20567] | 4257 | 0.132 | 0.4495 | No |
| 18 | CMPK1 | cytidine/uridine monophosphate kinase 1 [Source:HGNC Symbol;Acc:HGNC:18170] | 5408 | 0.039 | 0.3645 | No |
| 19 | DLG1 | discs large MAGUK scaffold protein 1 [Source:HGNC Symbol;Acc:HGNC:2900] | 6219 | -0.022 | 0.3041 | No |
| 20 | NME1 | NME/NM23 nucleoside diphosphate kinase 1 [Source:HGNC Symbol;Acc:HGNC:7849] | 6328 | -0.031 | 0.2992 | No |
| 21 | PPIP5K1 | diphosphoinositol pentakisphosphate kinase 1 [Source:HGNC Symbol;Acc:HGNC:29023] | 6621 | -0.051 | 0.2822 | No |
| 22 | AK6 | adenylate kinase 6 [Source:HGNC Symbol;Acc:HGNC:49151] | 6824 | -0.065 | 0.2738 | No |
| 23 | IP6K2 | inositol hexakisphosphate kinase 2 [Source:HGNC Symbol;Acc:HGNC:17313] | 7584 | -0.126 | 0.2290 | No |
| 24 | IP6K1 | inositol hexakisphosphate kinase 1 [Source:HGNC Symbol;Acc:HGNC:18360] | 7948 | -0.151 | 0.2177 | No |
| 25 | AK4 | adenylate kinase 4 [Source:HGNC Symbol;Acc:HGNC:363] | 9682 | -0.290 | 0.1157 | No |
| 26 | TJP2 | tight junction protein 2 [Source:HGNC Symbol;Acc:HGNC:11828] | 9772 | -0.298 | 0.1423 | No |
| 27 | ITPKA | inositol-trisphosphate 3-kinase A [Source:HGNC Symbol;Acc:HGNC:6178] | 10450 | -0.367 | 0.1309 | No |
| 28 | ITPKC | inositol-trisphosphate 3-kinase C [Source:HGNC Symbol;Acc:HGNC:14897] | 11674 | -0.528 | 0.0952 | No |
Table: GSEA details [plain text format]

  

Fig 2: GOMF\_PHOSPHOTRANSFERASE\_ACTIVITY\_PHOSPHATE\_GROUP\_AS\_ACCEPTOR      
 Blue-Pink O' Gram in the Space of the Analyzed GeneSet

  

Fig 3: GOMF\_PHOSPHOTRANSFERASE\_ACTIVITY\_PHOSPHATE\_GROUP\_AS\_ACCEPTOR: Random ES distribution      
 Gene set null distribution of ES for **GOMF\_PHOSPHOTRANSFERASE\_ACTIVITY\_PHOSPHATE\_GROUP\_AS\_ACCEPTOR**
